# Dynasore protects the ocular surface against damaging oxidative stress

**DOI:** 10.1371/journal.pone.0204288

**Published:** 2018-10-10

**Authors:** Andrew Webster, Shravan K. Chintala, Jasmine Kim, Michelle Ngan, Tatsuo Itakura, Noorjahan Panjwani, Pablo Argüeso, Joseph T. Barr, Shinwu Jeong, M. Elizabeth Fini

**Affiliations:** 1 USC Institute for Genetic Medicine, Keck School of Medicine of USC, University of Southern California, Los Angeles, CA, United States of America; 2 Program in Biological Sciences, USC Dana and David Dornsife College of Letters, Arts and Sciences, University of Southern California, Los Angeles, CA, United States of America; 3 Program in Preventive Medicine, Keck School of Medicine of USC, University of Southern California, Los Angeles, CA, United States of America; 4 New England Eye Center/Department of Ophthalmology and Department of Developmental, Molecular and Chemical Biology, Tufts University School of Medicine, Boston, MA, United States of America; 5 Schepens Eye Research Institute of Massachusetts Eye and Ear, Department of Ophthalmology, Harvard Medical School, Boston, MA, United States of America; 6 The Ohio State University College of Optometry, Columbus, OH, United States of America; 7 USC Institute for Genetic Medicine and USC Roski Eye Institute/Department of Ophthalmology, Keck School of Medicine of USC, University of Southern California, Los Angeles, CA, United States of America; Cedars-Sinai Medical Center, UNITED STATES

## Abstract

“Vital” dyes such as fluorescein and rose bengal are used clinically to evaluate ocular surface health; however, staining mechanisms remain poorly understood. Recent evidence suggests that sublethal cell damage stimulates fluorescein dye uptake. Since damage can also stimulate reparative plasma membrane remodeling, we hypothesized that dye uptake occurs via endocytic vesicles. Using an oxidative stress model, we show that damage to relatively undifferentiated monolayer cultures of human corneal epithelial cells stimulates uptake of fluorescein and rose bengal dyes and also stimulates endocytosis. Importantly, dye uptake was blocked by co-treatment with three different endocytosis inhibitors. Damage to stratified and differentiated corneal epithelial cell cultures, which are a better model of the ocular surface, also stimulated dye uptake; however, endocytosis was *not* stimulated in this case, and two of the inhibitors did not block dye uptake. The exception was the inhibitor Dynasore and its more potent analogue Dyngo-4a, small molecules that target dynamin family GTPases, but also have off-target effects on the plasma membrane. Significantly, while Dynasore blocked stress-stimulated dye uptake at the ocular surface of *ex vivo* mouse eyes when treatment was performed at the same time as eyes were stressed, it had no effect when used *after* stress was applied and the ocular surface was already damaged. Thus, Dynasore could not be working by inhibiting endocytosis. Employing cytotoxicity and western blotting assays, we demonstrate an alternative mechanism, showing that Dynasore is remarkably protective of cells and their surface glycocalyx, preventing damage due to oxidative stress, and thus precluding dye entry. These unexpected and novel findings provide greater insight into mechanisms of vital dye uptake and emphasize the importance of using a differentiated cell culture model for such studies. They also suggest that Dynasore and analogues might be used therapeutically to protect the ocular surface and to treat ocular surface disease.

## Introduction

The wet ocular surface comprises the stratified squamous mucosal epithelia of the cornea/conjunctiva and the overlying tear film [1]. These cells are continually renewed in a process whereby daughter cells generated by division of basal cells at the basement membrane are displaced upward in the cell layers, at the same time undergoing terminal differentiation. Cells in the apical cell layer are morphologically and biochemically very different, from cells in the basal layer. As they approach the surface, cells increasingly flatten and begin to express mucosal markers in a polarized manner, including membrane-associated mucins such as MUC16, that emanate from specialized membrane folds on the apical cell layer called microplicae. MUC16 binds multiple oligomers of the galectin LGALS3 to form a highly organized glycocalyx [2]. The glycocalyx, along with the plasma membranes themselves, creates a transcellular barrier to prevent intracellular penetration [2, 3]. Tight junctions seal the space between adjacent cells to create a paracellular barrier, preventing penetration into deeper cell layers [4]. As the apical cells mature further, their surface areas increase, their microplicae flatten, and MUC16 is lost from their surfaces [5]. In addition, the cells become less active metabolically, ultimately being shed in a form of cell death called desquamation [6]. In humans, complete turnover of the ocular surface epithelia occurs in 5–7 days [7, 8].

Directly exposed to the external environment, the ocular surface epithelia are subject to damaging agents and physical insults such as ultraviolet light, microorganisms and allergens, that cause ocular surface damage, barrier disruption and increased desquamation [1]. Ocular surface damage is characteristic of dry eye disease (keratoconjunctivitis sicca), a desiccating condition of the ocular surface affecting 20% or more of the population in North America, Europe, and Asia [9]. The most commonly used method for tracking ocular surface damage due to such challenges is staining with water soluble “vital” dyes [10]. Fluorescein was first used clinically in 1882 for evaluation of corneal epithelial defects [11]. Rose bengal use was popularized in the 1930s for dry eye diagnosis because of the distinctive “punctate” staining pattern observed at the ocular surface of patients [12]. Fluorescein is now used for this purpose as well [13]. Exposure to multipurpose contact lens cleaning solutions (MPS) also causes staining with vital dyes, a recently recognized phenomenon called solution-induced corneal staining (SICS) [14].

Considering the widespread use of vital dyes, it is surprising that the mechanism of staining is still not well understood [13]. Studies published in the early 1990s reported that healthy cells in monolayer culture take up rose bengal [15] and that tear components such as mucins block uptake. Later it was shown that corneal epithelial cells in culture exclude rose bengal autonomously if induced to differentiate and elaborate a mucosal glycocalyx [1]. This suggested that punctate staining with rose bengal in dry eye may represent damage to the glycocalyx barrier of individual cells, allowing dye to penetrate. It also was the first indication that relatively undifferentiated monolayer cell cultures cannot provide a complete model of the ocular surface.

The hydroxyxanthine, fluorescein, is the parent compound from which rose bengal was derived, thus, the two dyes are structurally related [16]. Nevertheless, they differ in cell uptake properties. Living corneal epithelial cells in monolayer culture take up fluorescein in the same way as rose bengal, but at a lower level requiring visualization under epifluorescent illumination [17]. Fluorescein uptake by individual corneal epithelial cells was also observed at the rabbit ocular surface *in situ* under epifluorescent illumination [18]. In later studies, individual cells in the superficial epithelial layers of the human ocular surface damaged by dry eye were observed to take up fluorescein, described as “hyperstaining” [19]. Unlike rose bengal, fluorescein uptake by cells is not blocked by mucins [17], excluding glycocalyx damage as the cause of hyperstaining. This means that the plasma membrane must be the primary barrier to dye penetrance.

Two recent studies used cells in monolayer culture to investigate basic mechanisms of fluorescein uptake [20, 21]. While all cells took up fluorescein at a low level as previously reported [17], a small percentage were observed to concentrate dye, thus standing out as hyperfluorescent. Fluorescein concentration was observed to be an active process, inhibited by reducing the temperature or by killing the cells [21]. Application of a damaging stress [20], or treatment with an MPS [21], greatly increased the number of hyperfluorescent cells. Stressed cells exhibiting high fluorescence intensity also showed characteristics of early apoptosis, whether in monolayer culture, or in the apical epithelial layer of ex vivo rabbit eyes [20]. These findings suggest that fluorescein hyperstaining is an active process of dye concentration in cells that may be compromised, but which are still living.

When the cell surface is compromised, it may respond by activating remodeling processes to repair plasma membrane damage and maintain proteostasis [22–25]. We hypothesized that fluorescein dye might be taken up into endocytic vesicles of individual cells undergoing such repair. Here we report our investigation of this idea using undifferentiated human corneal epithelial cells in monolayer culture, stratified and differentiated cultures, and mouse corneas *ex vivo*, in order to observe the full range of factors that might affect fluorescein uptake, and we include a comparison to rose bengal dye, which enables assessment of cell differentiation.

## Materials and methods

HUGO nomenclature is used for genes and their products throughout the text.

### Experimental model, study design and statistical analysis

The purpose of this study was to investigate mechanisms of dye uptake relevant to ocular surface disease. We hypothesized that stimulation of plasma membrane remodeling by sublethal cell damage would result in vital dye uptake into endocytic repair vesicles. Oxidative stress has been implicated in ocular surface damage due to dry eye [26] and many other ocular surface disorders [27, 28], thus we chose this as the damaging stress for our study. An aqueous stock solution of tert-butyl hydroperoxide (tBHP) was diluted into the medium of human cell cultures or mouse eye organ cultures as we have previously described [29]. The final dilution of tBHP used for cell culture experiments was 3 mM or 10 mM (as indicated in the individual experimental details). A final dilution of 10 mM tBHP was used for mouse eye organ culture experiments. These optimal concentrations were determined empirically in dose-response assessments as causing a high level of vital dye staining without killing cells. As soon as tBHP was added, all cultures were returned to the cell culture incubator under 5% CO_2_ atmosphere. Stressed cultures were compared to unstressed controls incubated similarly for the same time period. Cells were incubated for 2 to 3 hours and mouse eyes were incubated for 2.5 hours before endpoint determination.

To learn whether endocytosis was involved in dye uptake, the process was blocked by treating cells or eyes with endocytic inhibitors. Chlorpromazine hydrochloride (MP Biologicals, Solon, OH), an inhibitor of clathrin-mediated endocytosis [30], was used at a final dilution of 8 ug/mL. Genistein (Sigma-Aldrich, St Louis, MO), an inhibitor of caveolin-mediated endocytosis [30], was used at 200 uM final dilution. Dynasore hydrate (Sigma-Aldrich, St. Louis, MO), a GTPase inhibitor that rapidly and reversibly inhibits the activity of dynamin family members involved in both clathrin- and caveolin-mediated endocytosis [31], was diluted to a final concentration of 40 uM or 80 uM (as indicated in the individual experimental details). The Dynasore derivative Dyngo-4a (Abcam, Cambridge, UK), a more potent dynamin inhibitor [32], was used at 15 uM final dilution. All inhibitors were dissolved in DMSO, which served as the treatment vehicle. The same volume of DMSO was added to matching untreated cultures as a vehicle control.

At the end of an experiment, the vital dye assay and/or other assays, were performed on the cells, conditioned culture medium, or eyes immediately.

All data are shown as the mean ± standard deviation (SD). All assays were performed in triplicate (n = 3). The statistical significance of two data sets was assessed by the Student’s *t* test. For the calculation of *P* values, all technical replicates from all biological replicates were used. Statistical significance was determined at *P* < 0.05. Individual experiments were repeated at least twice.

### Human corneal epithelial cell culture

A telomerase-immortalized line of human corneal limbal epithelial (HCLE) cells was used for all experiments employing cell culture [33, 34]. The cell line was developed in the Gipson lab [33] according to methods described [35], and authenticated by marker expression analysis [2] and by chromosomal analysis and use of polymorphic short tandem repeat (STR) loci [36]. The cell line was derived from normal tissue and expresses the same mucin gene and keratin repertoire as native epithelia when stimulated to differentiate [33]. For an experiment, cells were plated in 96-well plates and used at 90% confluence as monolayers, or transferred to differentiation medium containing high calcium ion and bovine serum and left for 7 days to stratify and differentiate, as described [33]. Stratification was routinely evaluated using phase contrast microscopy; differentiation leading to glycocalyx barrier function was evaluated using the rose bengal penetration assay. Results of both of these assays have been previously shown [34].

### Mouse eye organ culture

The University of Southern California’s Institutional Animal Care and Use Committee approved the research protocol number 11412 for use of mice in this study. Research was conducted in adherence with the Association for Research in Vision and Ophthalmology (ARVO) Statement for the Use of Animals in Ophthalmic and Visual Research. Wild type C57Bl/6J mice, 6–8 weeks of age, were purchased from Jackson Labs (Bar Harbor, ME). Prior to use in an experiment, mice were housed in a pathogen-free barrier facility and kept at 25±1^°^C, relative humidity 60%±10%, with alternating 12-hour light/dark cycles. To obtain eyes for organ culture, euthanasia was performed using compressed CO_2_ gas, according to the American Veterinary Medical Association Guidelines for the Euthanasia of Animals: 2013 Edition. Eyes were enucleated immediately, washed in PBS, and then placed in Keratinocyte-SFM (K-SFM) media (Thermo Fisher Scientific, Waltham, MA) [33].

### Vital dye staining assay

Vital dye staining of cells in culture was performed using sodium fluorescein (Sigma-Aldrich). Staining of mouse eyes was performed using a clinical fluorescein dye solution (Fluoresoft-0.35%, Holles Laboratories, Cohasset, MA). The two dyes provide similar staining results [37, 38]. Rose bengal (0.05%; Sigma-Aldrich, St. Louis, MO) was used for both cells in culture and mouse eyes. Vital dyes were added to cultures for 10 minutes and then excess dye was removed by washing 3 times with PBS. Dye uptake was evaluated qualitatively by imaging. Images (excitation/emission = 488/510 nm) were taken immediately after washing, so that staining could be evaluated before dye diffused. Fluorescein uptake by cultured cells was quantified using a plate reader and depicted in graphs as relative fluorescence units (RFU). Rose bengal uptake by cultured cells or eyes was quantified using a modification of a previously described method [37]. After imaging, the stained cells or eyes were incubated in 100 uL of DMSO at room temperature for 1 hour, and the solution was recovered into the wells of a 96 well plate to read in a plate reader at 562 nm.

### Apoptosis assays

Early apoptosis was assessed by probing both stratified and monolayer HCLE cultures with Alexa Fluor 594-conjugated Annexin-5 (Invitrogen, Molecular Probes, Eugene, OR). The manufacturer’s protocol was followed, with some modification. Thus, instead of harvesting the cells after induction, the ANXA5 conjugate was applied directly to the wells at 25 uL per 100 uL of media. The cells were then incubated in the dark at room temperature for 15 minutes and imaged with a Keyence BZ-X700 fluorescence microscope (excitation/emission = 532/588 nm).

Late apoptosis was assessed with the In Situ Cell Death Detection Kit, Fluorescein, (Roche, Basel, Switzerland). Cells were washed with PBS and incubated in terminal deoxynucleotide transferase dUTP nick end-labeling (TUNEL) reaction mixture for 1 hour at 37°C in the dark, following the supplier’s protocol, and then washed three time with PBS. Images were taken using a Keyance BZ-X700 fluorescence microscope (excitation/emission = 488/510 nm).

### Endocytosis assay

Endocytosis was quantified by monitoring cell uptake of human Alexa Fluor 568-conjugated TF (serum transferrin; Thermo Fisher Scientific, Waltham, MA). Monolayer or stratified cultures of HCLE cells were washed once with PBS, and then again with basal K-SFM media. Cells were imaged by phase contrast to ensure that all wells were of equal cell density. Then cells were probed with Alexa Fluor 568-conjugated TF using the manufacturer’s protocol with some modifications. Instead of putting the cells on ice and washing with cold Living Cell Imaging Solution (LCIS), cells were washed with basal K-SFM media. Cells were probed with 10x Alexa Fluor 568-conjugated TF (250 ug/mL) diluted in LCIS for 20 minutes before being washed in cold LCIS. Cells were then imaged with a Keyence BZ-X700 fluorescence microscope (excitation/emission = 532/588 nm). Alexa Fluor 568-conjugated TF uptake was quantified by image J analysis.

### Metabolic assays for cytotoxicity

Two commercial metabolic assays were used to assess cytotoxicity: MTT (3-[4,5-dimethylthiazol-2-yl]-2,5-diphenyl tetrazolium bromide; Thermo Fisher Scientific) or WST-1 (4-[3-(4-iodophenyl)-2-(4-nitrophenyl)-2H-5-tetrazolio]-1,3-benzene disulfonate; Thermo Fisher Scientific). Superoxide anions generated by NAD(P)H-dependent cellular oxidoreductase activity reduce MTT or WST to water-soluble formazans which absorb visible light [39]. MTT dye is cell permeable and reduction occurs intracellularly; WST-1 dye is cell-impermeable thus, reduction occurs outside the cell via plasma membrane electron transport [40]. Dye reduction was measured by absorbance at wavelengths of 590 nm (MTT) or 440 nm (WST-1).

### Trypan blue exclusion assay

The trypan blue exclusion assay is based on the principle that live cells possess intact cell membranes that exclude certain dyes, whereas dead cells do not exclude the dye [41]. Cells were stained with filtered 0.4% trypan blue dye (Thermo Fisher Scientific) for 4 minutes following 3 washes with PBS. The extent of staining was measured qualitatively by imaging under white light, and quantitatively using a plate reader at 590 nm.

### Lectin binding

To determine the presence of mucin-type glycoconjugates on cell surfaces, a binding assay was performed with jacalin, a lectin that specifically recognizes the T-antigen present on O-glycans [42, 43]. Methods were as previously described [34]. Briefly, cells fixed in 100% methanol were incubated in blocking buffer (1% BSA in PBS) for 30 minutes. Cultures were then incubated with fluorescein-conjugated jacalin (Vector Lab, Burlingame, CA) at a dilution of 1:100 for 1 hour at room temperature, washed in PBS, then cover-slipped and images were taken using a Keyance BZ-X700 fluorescence microscope (excitation/emission = 488/510 nm).

### Western blotting

Proteins from equal volume cell culture media samples were separated by SDS-PAGE and transferred to polyvinylidene difluoride (PVDF) membranes (Thermo Fisher Scientific). Membranes were probed with a primary antibody against LGALS3 (sc-23983; Santa Cruz Biotech, Santa Cruz, CA) overnight (at 1:200 dilution) at 4^°^C with gentle shaking. Membranes were then incubated for 1 hour with secondary antibody–horseradish peroxidase conjugates (Santa Cruz Biotechnology, Santa Cruz, CA) at a dilution of 1:10,000. Specific signals were developed for 1 min using the enhanced chemiluminescence (ECL) kit components 1 and 2 (GE Healthcare UK Limited, Buckinghamshire, UK). Chemiluminescence was visualized by exposure of photographic film (LAS-4000; Fujifilm, Tokyo, Japan).

### Polymerase chain reaction

As a measure of cell lysis, cell culture medium was collected and an equal portion of the media was subjected to polymerase chain reaction (PCR) using primer sets for detection of ACTB gene fragments (forward primer: 5′-cattgccgacaggatgcaga-3′; reverse primer: 5′-ctgatccacatctgctggaa-3′), as described previously [44].

## Results

### Oxidative stress stimulates vital dye uptake linked with sublethal cell damage

**Fig 1:**
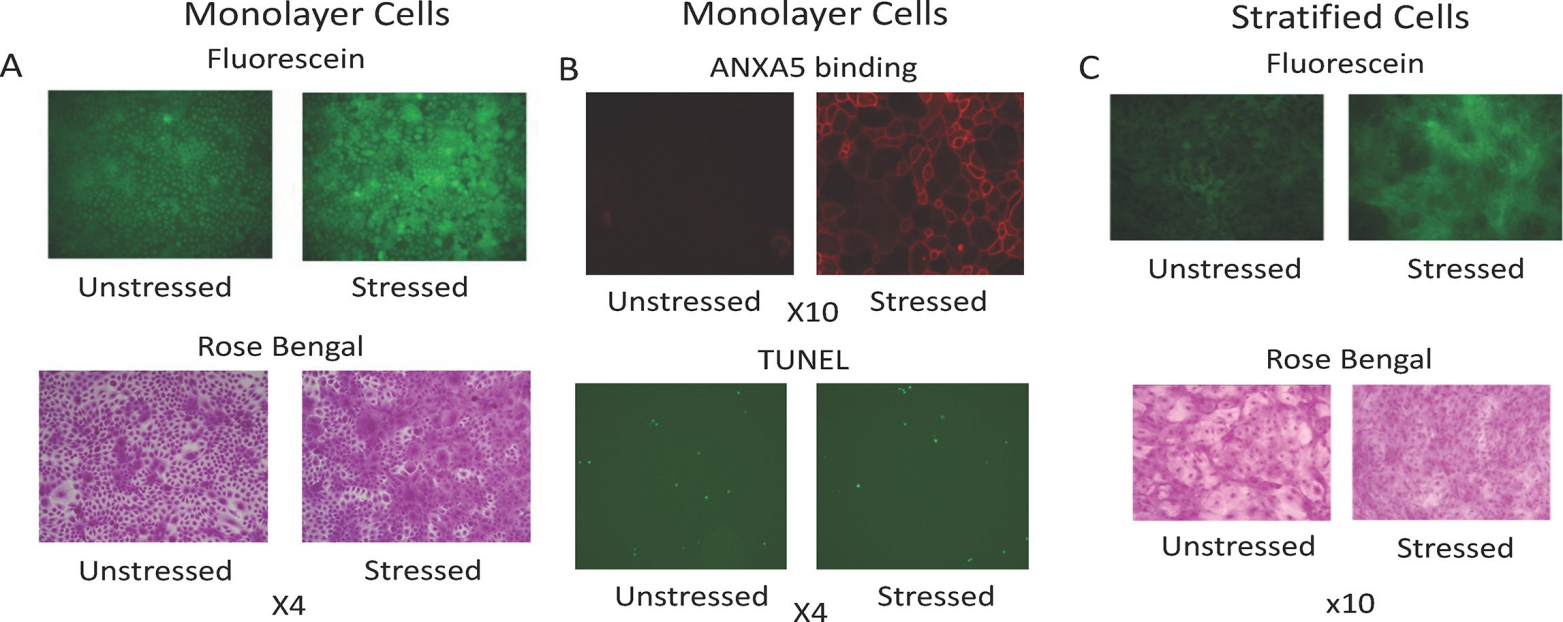
Oxidative stress stimulates vital dye uptake linked with sublethal cell damage. **A)** HCLE monolayer cell cultures plated in triplicate were either left unstressed or stressed with 3 mM tBHP. After 2 hours, cultures were stained with fluorescein or rose bengal and imaged under epifluorescent illumination (fluorescein) or white light (rose bengal). Representative images are shown from each set.**B)** HCLE monolayer cultures plated in triplicate were either left unstressed or stressed with 3 mM tBHP. After 2 hours, the ANXA5 binding assay or the TUNEL assay was performed to evaluate apoptosis. Representative images are shown from each set.**C)** HCLE stratified cell cultures plated in triplicate were either left unstressed or stressed with 3 mM tBHP. After 2 hours, cultures were stained with fluorescein or rose bengal and imaged under epifluorescent illumination (fluorescein) or white light (rose bengal). Representative images from each set are shown from each set.

The goal of our first set of experiments was to characterize and validate our oxidative stress model in monolayer cultures of human corneal epithelial cells, referencing the two recent studies discussed in the Introduction [20, 21]. Representative results are shown in Fig 1.

As visualized under epifluorescent illumination (Fig 1A), fluorescein stained all cells in monolayer culture, with dye concentrated primarily in the nucleus, much as described in the original reports [15, 17]. A mosaic of scattered individual hyperstained cells was observed, similar to both of the reference studies [20, 21]. Visual inspection revealed that oxidative stress increased the number of cells hyperstained by fluorescein, again consistent with both of the reference studies [20, 21]. Rose-bengal dye uptake was essentially the same as fluorescein, with similar numbers of cells showing dye concentration under both unstressed and stressed conditions, as judged by visual inspection (Fig 1A). In agreement with the report of Bakkar and colleagues [20], fluorescein dye uptake (as quantified by plate reader) stimulated by oxidative stress was inhibited to 35% when the culture temperature was reduced to ambient, and to 10% when reduced to 4°C (data not shown). This is consistent with the idea that vital dye uptake and concentration is an active process of living cells.

To characterize cell damage caused by oxidative stress, we probed for both early (ANXA5 binding assay) and late (TUNEL assay) stages of apoptosis. Phosphatidylserine exposed on the outer leaflet of the membrane surface leads to ANXA5 binding. The percentage of cells that bound ANXA5 was substantially increased by oxidative stress, as judged by visual inspection (Fig 1B). This agrees with the findings of one of the reference studies [20]. In contrast, only a small number of cells appeared to be in late stage apoptosis was detected by TUNEL assay performed at the same time point, and there was little if any difference between unstressed and stressed cells (Fig 1B). This supports the idea that cell damage due to oxidative stress in our model is primarily sublethal, at least at the time point examined.

Next, we attempted to determine whether oxidative stress would also stimulate hyperstaining in stratified and differentiated cell cultures (Fig 1C). To assess differentiation, cultures were stained with rose bengal to measure glycocalyx barrier function. Differentiated cells pile up in mounds, distinguished by their pale rose to white color in a sea of darkly-stained cells. Oxidative stress greatly reduced the area occupied by white islands in rose bengal-stained cultures and also increased the amount of staining with fluorescein dye (as judged by visual inspection). However, the appearance of individual cells with dye accumulated in the cytoplasm, as seen in monolayer cultures, was not evident in these stratified and differentiated cultures.

### Endocytosis inhibitors block oxidative stress-stimulated vital dye uptake in monolayer cells

**Fig 2:**
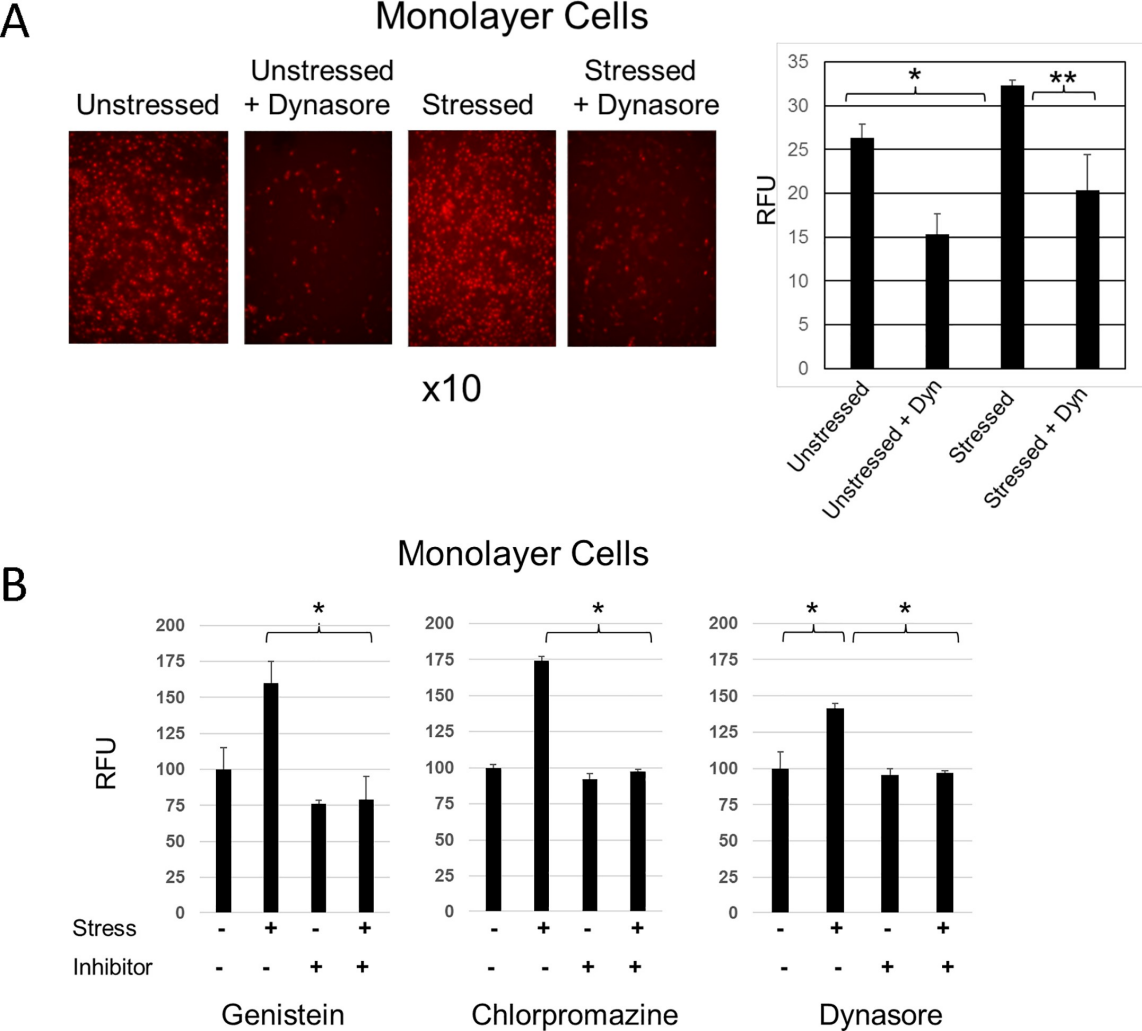
Endocytosis inhibitors block oxidative stress-stimulated vital dye uptake in monolayer cells. **A)** HCLE monolayer cultures plated in triplicate were either left unstressed or stressed with 3 mM tBHP. A parallel set of cultures were treated with Dynasore (40 uM). (An equal volume of the Dynasore diluent, DMSO, was added to untreated cultures.) Alexa Fluor 568-conjugated TF, internalization of which is a marker of the endocytic process, was added to all cultures at the beginning of an experiment. The fluorescein density in individual images was measured using Image J software: *, p<0.01; **, p<0.05 (student t-test, n = 3). **B)** HCLE monolayer cell cultures plated in triplicate were either left unstressed or stressed with 3 mM tBHP. At the same time, a parallel set of cultures were treated with one of three different endocytosis inhibitors: Genistein (200 uM), Chlorpromazine (8 ug/mL) or Dynasore (40 uM). (An equal volume of the inhibitor diluent, DMSO, was added to untreated cultures.) After 2 hours, cultures were stained with fluorescein and staining was quantified using a plate reader and depicted as RFU (relative fluorescence unit). The effect of the inhibitors on dye uptake was evaluated for statistical significance: *, p<0.01 (student t-test, n = 3).

Next, we investigated the possible role of endocytosis in oxidative stress-stimulated vital dye uptake by monolayer cell cultures. Representative results are shown in Fig 2.

First, we determined whether endocytosis was stimulated by oxidative stress. Cells were stressed in the presence of Alexa Fluor 568-conjugated TF, internalization of which is a marker of the endocytic process. As predicted, oxidative stress-stimulated TF uptake by cells in monolayer culture (Fig 2A). Co-treatment with Dynasore, which inhibits dynamin GTPases necessary for TF endocytosis, blocked stress-stimulated TF uptake.

Next, we determined whether endocytosis inhibitors blocked stress-stimulated vital dye uptake. Stress-stimulated fluorescein dye uptake was blocked by genistein, an inhibitor of caveolin-mediated endocytosis, and chlorpromazine, an inhibitor of clathrin-mediated endocytosis. It was also blocked by Dynasore, which is required for both forms of endocytosis (Fig 2B).

These results link between stress-stimulated endocytosis and vital dye uptake in monolayer cell cultures.

### Only Dynasore and its analogue Dyngo-4a block oxidative stress-stimulated vital dye uptake in stratified and differentiated cells

**Fig 3:**
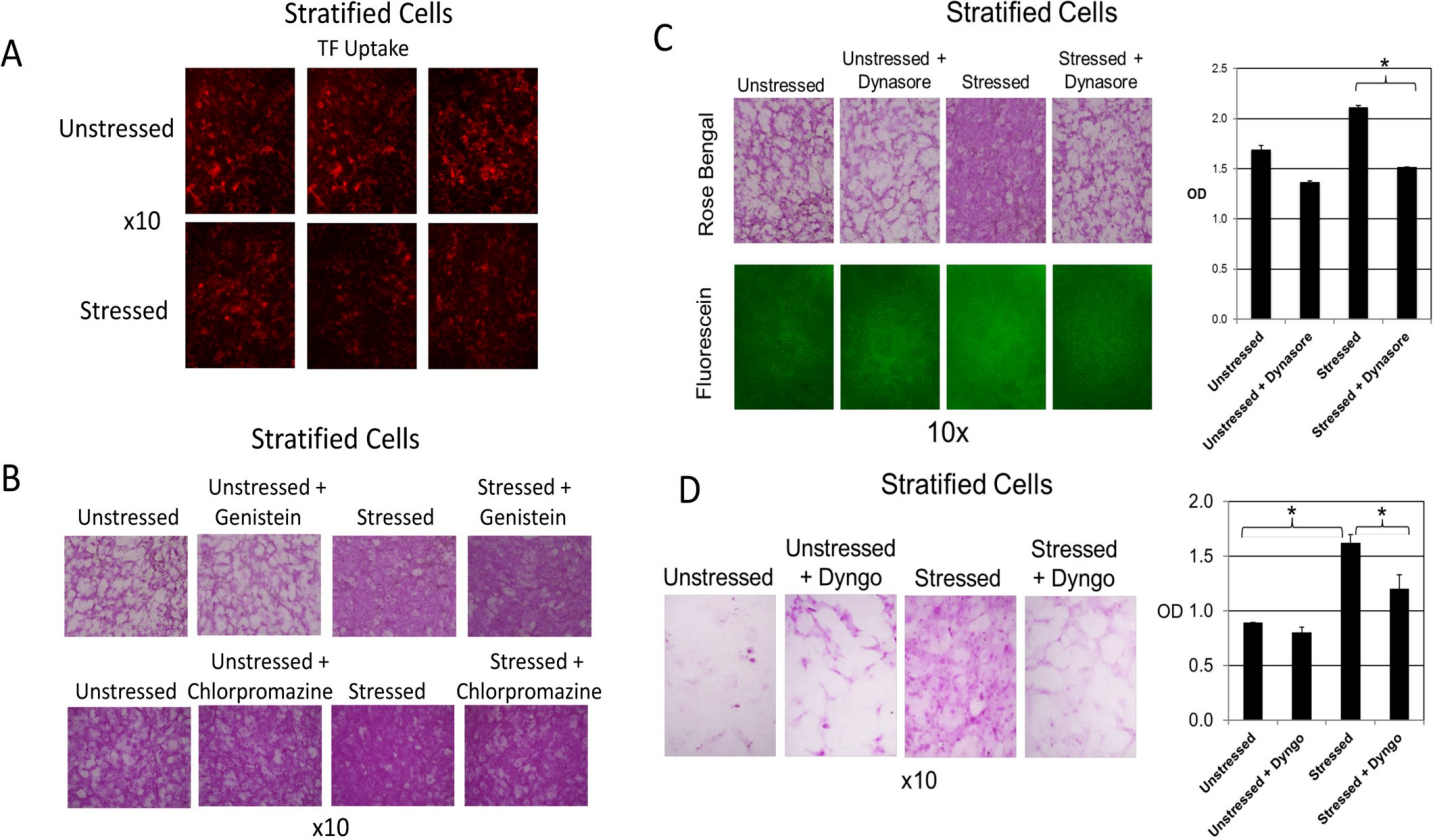
Only Dynasore and its analogue Dyngo-4a block oxidative stress-stimulated vital dye uptake in stratified cells. **A)** HCLE stratified cultures plated in triplicate were either left unstressed or stressed with 10 mM tBHP. Alexa Fluor 568-conjugated TF, internalization of which is a marker of the endocytic process, was added to all cultures at the beginning of an experiment. Images are shown for each triplicate set.**B)** HCLE stratified cultures plated in triplicate were either left unstressed or stressed with 10 mM tBHP. At the same time, a set of unstressed and stressed cultures were treated with Genistein (200 uM). (An equal volume of the Dynasore diluent, DMSO, was added to untreated cultures.) After 2 hours, cultures were stained with rose bengal. A similar experiment was done using Chlorpromazine (8 ug/mL). Representative images are shown from each triplicate set.**C)** HCLE stratified cultures plated in triplicate were either left unstressed or stressed with 10 mM tBHP. At the same time, a parallel set of unstressed and stressed cultures were treated with Dynasore (40 uM). (An equal volume of the Dynasore diluent, DMSO, was added to untreated cultures.) After 3 hours, cultures were stained with fluorescein or rose bengal and imaged under epifluorescent illumination (fluorescein) or white light (rose bengal). Representative images are shown from each triplicate set. Quantification of rose bengal staining was performed: *, p<0.01 (student t-test, n = 3). OD = optical density at 562 nm.**D)** HCLE stratified cultures plated in triplicate were either left unstressed or stressed with 10 mM tBHP. At the same time, a set of unstressed and stressed cultures were treated with Dyngo-4a (15 uM). (An equal volume of the Dynasore diluent, DMSO, was added to untreated cultures.) After 3 hours, parallel sets of cultures were stained with fluorescein or rose bengal and imaged under epifluorescent illumination (fluorescein) or white light (rose bengal). Representative images are shown from each triplicate set. Quantification of rose bengal staining was performed: *, p<0.01(student t-test, n = 3).

In our next set of experiments, we examined the possible connection between endocytosis and vital dye uptake by stratified and differentiated cell cultures. These experiments were designed similarly to those described above, using monolayer cell cultures. Representative results are shown in Fig 3.

First, we investigated whether endocytosis was stimulated by oxidative stress, as was done in the monolayer cell culture experiments described above. The results were quite different in stratified cell cultures. Oxidative stress did not stimulate TF uptake, in fact there appeared to be some inhibition (Fig 3A).

Next, we investigated the possible role of endocytosis in oxidative stress-stimulated vital dye uptake, as done for the monolayer cell culture experiments. Again, the result was quite different in stratified and differentiated cell cultures. Genistein and chlorpromazine had no effect on vital dye uptake (Fig 3B), in these cultures. Interestingly, Dynasore continued to be a very effective inhibitor (Fig 3C). Because of this we also tried the Dynasore analogue, Dyngo-4a, a more potent inhibitor of dynamin GTPases. Dyngo-4a was also a very effective inhibitor of vital dye uptake in stratified and differentiated cell cultures when used at a lower concentration (Fig 3D).

These results indicate that the link between vital dye uptake and endocytosis, observed in monolayer cells, does not hold for stratified and differentiated cells, which better model the ocular surface. The fact that Dynasore and its analogue Dyngo-4a inhibit vital dye uptake must thus occur through an alternative mechanism.

### Dynasore does *not* prevent oxidative stress-stimulated vital dye uptake after stress is applied and damage has occurred

**Fig 4:**
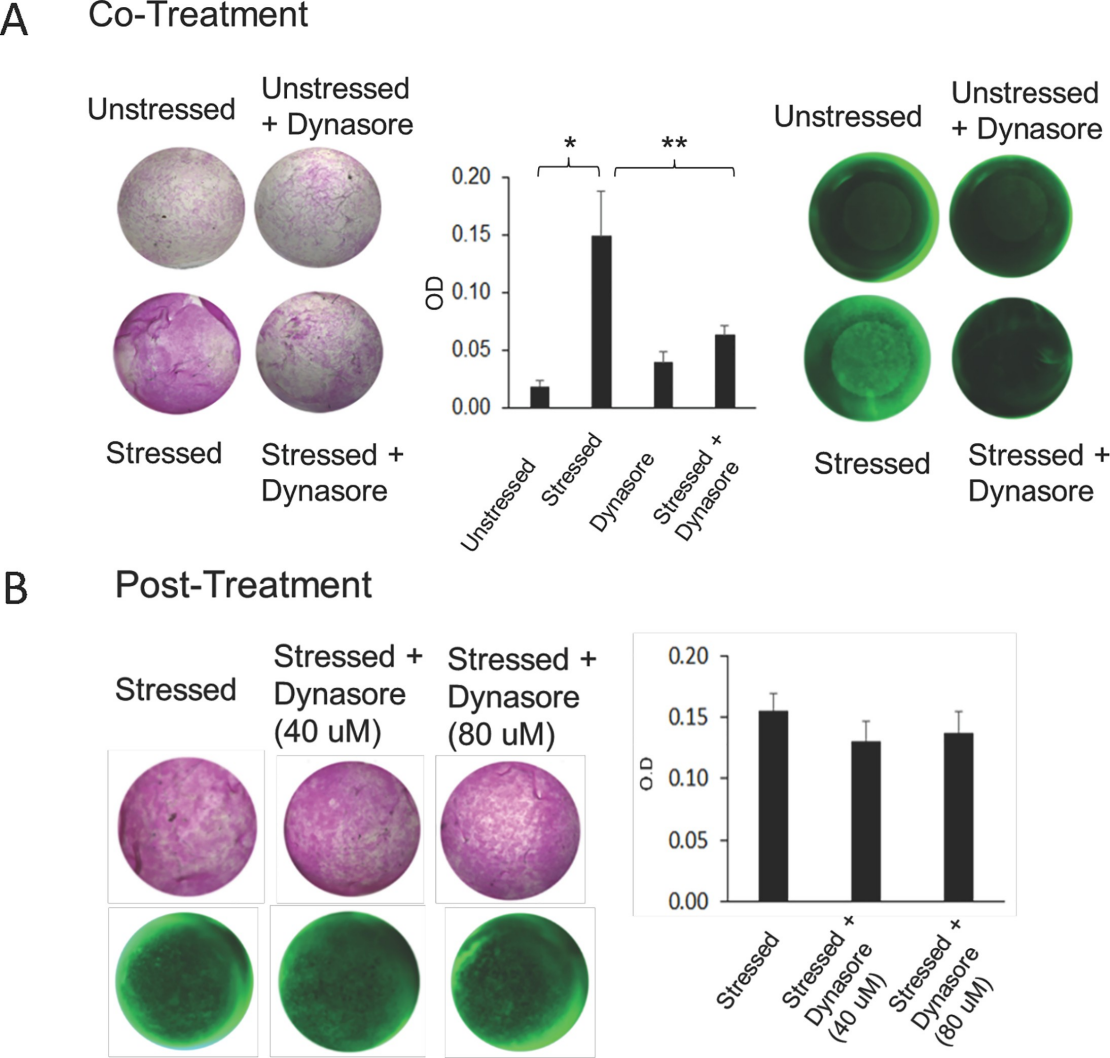
Dynasore does not prevent oxidative stress-stimulated vital dye uptake when used after stress is applied. A) Co-treatment. Mouse eyes, placed in organ culture in triplicate, were either left unstressed or stressed with 10 mM tBHP. At the same time, a set of unstressed and stressed eyes were treated with Dynasore (40 uM). (An equal volume of the Dynasore diluent, DMSO, was added to untreated cultures.) After 2 hours, parallel sets of cultures were stained with rose bengal or fluorescein and imaged under white light (rose bengal) or epifluorescent illumination (fluorescein). Representative images are shown from each triplicate set. Rose bengal staining was quantified using a plate reader. *, p<0.01; **, p<0.05 (student t-test, n = 3).**B) Post-treatment.** Mouse eyes placed in organ culture were first stressed with 10 mM tBHP for 2 hours, then triplicate sets were left untreated or treated with 40 or 80 uM Dynasore for 30 minutes. (An equal volume of the Dynasore diluent, DMSO, was added to untreated cultures.) Then, eyes were stained with rose bengal or fluorescein and imaged under white light (rose bengal) or epifluorescent illumination (fluorescein). Representative images are shown from each triplicate set. Rose bengal staining was quantified using a plate reader. (student t-test, n = 3).

As already noted, stratified cell cultures of corneal epithelial cells are a better model of the ocular surface than monolayer cultures. However, the stratified mounds of cells that develop in differentiation medium do not form a smooth and continuous layer like the corneal epithelium. The goal of the next set of experiments was to validate the cell culture findings made thus far to the actual ocular surface, and also to extend those findings. These experiments made use of the mouse eye organ culture model. Representative results are shown in Fig 4.

For the validation experiments, *ex vivo* mouse eyes were placed in organ culture and left unstressed or subjected to oxidative stress for three hours. At the end of this time period, eyes were removed from the culture medium and the vital dye staining assay was performed immediately. Application of stress stimulated a significant increase in dye uptake at the ocular surface (Fig 4A). Staining was fairly uniform, unlike dry eye, which manifests as punctate staining of individual cells. However, this type of uniform staining is characteristic of photokeratitis [45]. When eyes were treated with Dynasore at the same time as stress was applied, dye uptake was prevented. These results are consistent with the findings made using cells in culture.

To extend our investigation, we examined the effects of adding an endocytosis inhibitor *after* the eyes were subjected to oxidative stress. Dynasore is fast-acting, penetrating cells within seconds [46]. Nevertheless, we used both a 15-minute and a 30-minute post-treatment period, to ensure that Dynasore had time to work (Fig 4B shows the 30-minute time point). Significantly, dye uptake was *not* reduced, unlike the results when eyes were treated with Dynasore at the same time as stress was applied, even when the standard Dynasore concentration (40 uM) was doubled (80 uM).

These results provide further evidence to reject the hypothesis that stress stimulates vital dye uptake via endocytic vesicles.

### Dynasore protects the ocular surface subjected to damaging oxidative stress

**Fig 5:**
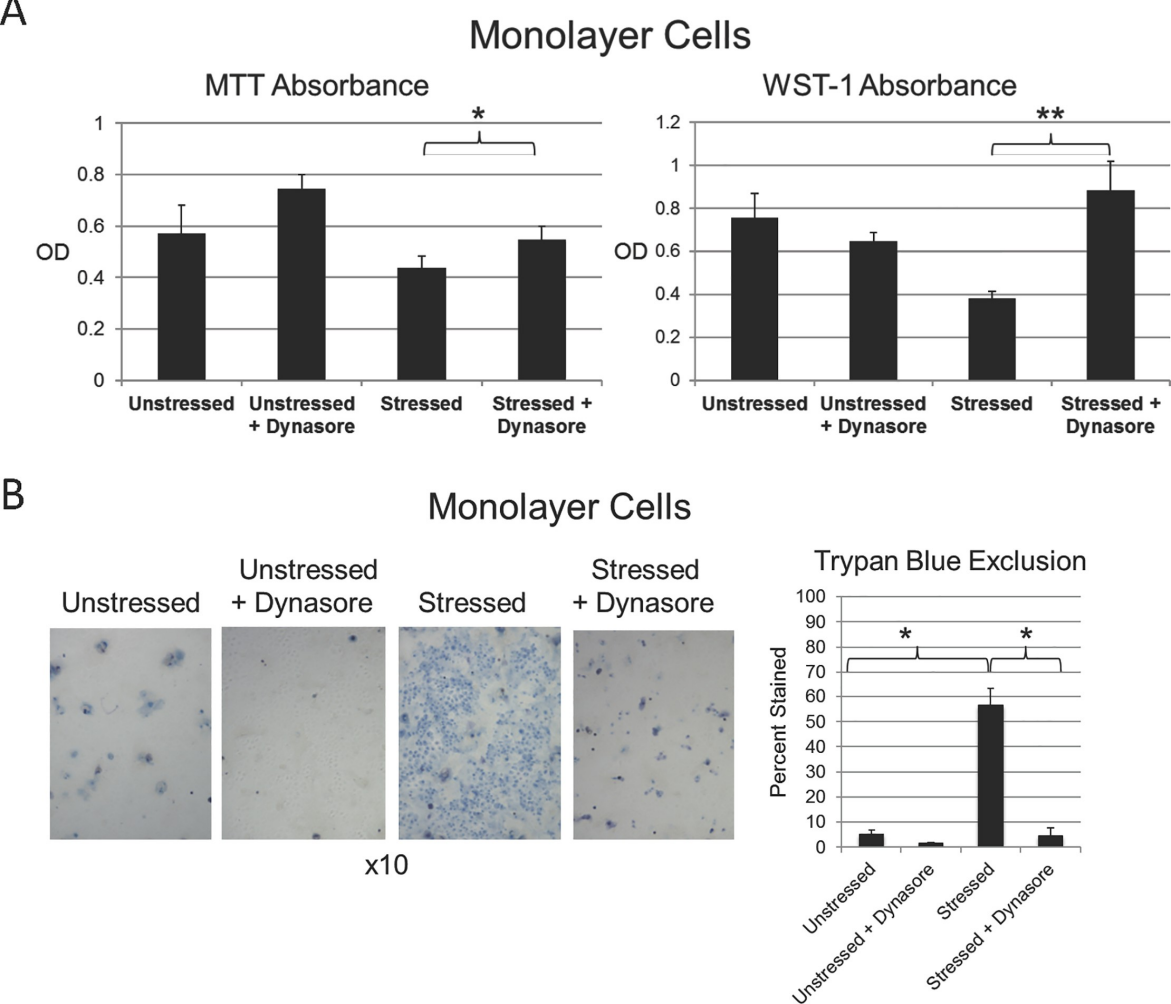
Dynasore protects cells against damage due to oxidative stress. **A)** HCLE monolayer cultures plated in triplicate were either left unstressed or stressed with 10 mM tBHP. At the same time, a set of unstressed and stressed cultures were treated with Dynasore (40 uM). (An equal volume of the Dynasore diluent, DMSO, was added to untreated cultures.) After 2 hours, WST-1 and MTT assays were performed: *, p<0.05; **, P<0.01 (student t-test, n = 3).**B)** HCLE monolayer cultures plated in triplicate were either left unstressed or stressed with 10 mM tBHP. At the same time, a set of unstressed and stressed cultures were treated with Dynasore (40 uM). (An equal volume of the Dynasore diluent, DMSO, was added to untreated cultures.) After 3 hours, cells were stained with trypan blue dye and imaged under white light. Representative images are shown from each triplicate set: *, p<0.01 (student t-test, n = 3).

The goal of the next set of experiments, was to investigate an alternative hypothesis, that Dynasore protects the ocular surface against damaging oxidative stress, thus precluding vital dye uptake. Representative results are shown in Fig 5.

First, we investigated protection of the cells themselves, using monolayer cultures. Applying the MTT/WST-1 metabolic assay, we found that absorbance was greatly increased by application of oxidative stress, indicative of cell damage. Treatment with Dynasore at the same time as stress was applied was protective, in particular for the WST-1 assay (Fig 5A). Applying the trypan blue exclusion assay for plasma membrane damage, we found that staining was greatly increased by application of oxidative stress, again indicative of cell damage. Treatment with Dynasore at the same time as stress was applied was remarkably protective, essentially eliminating trypan blue staining (Fig 5B).

**Fig 6:**
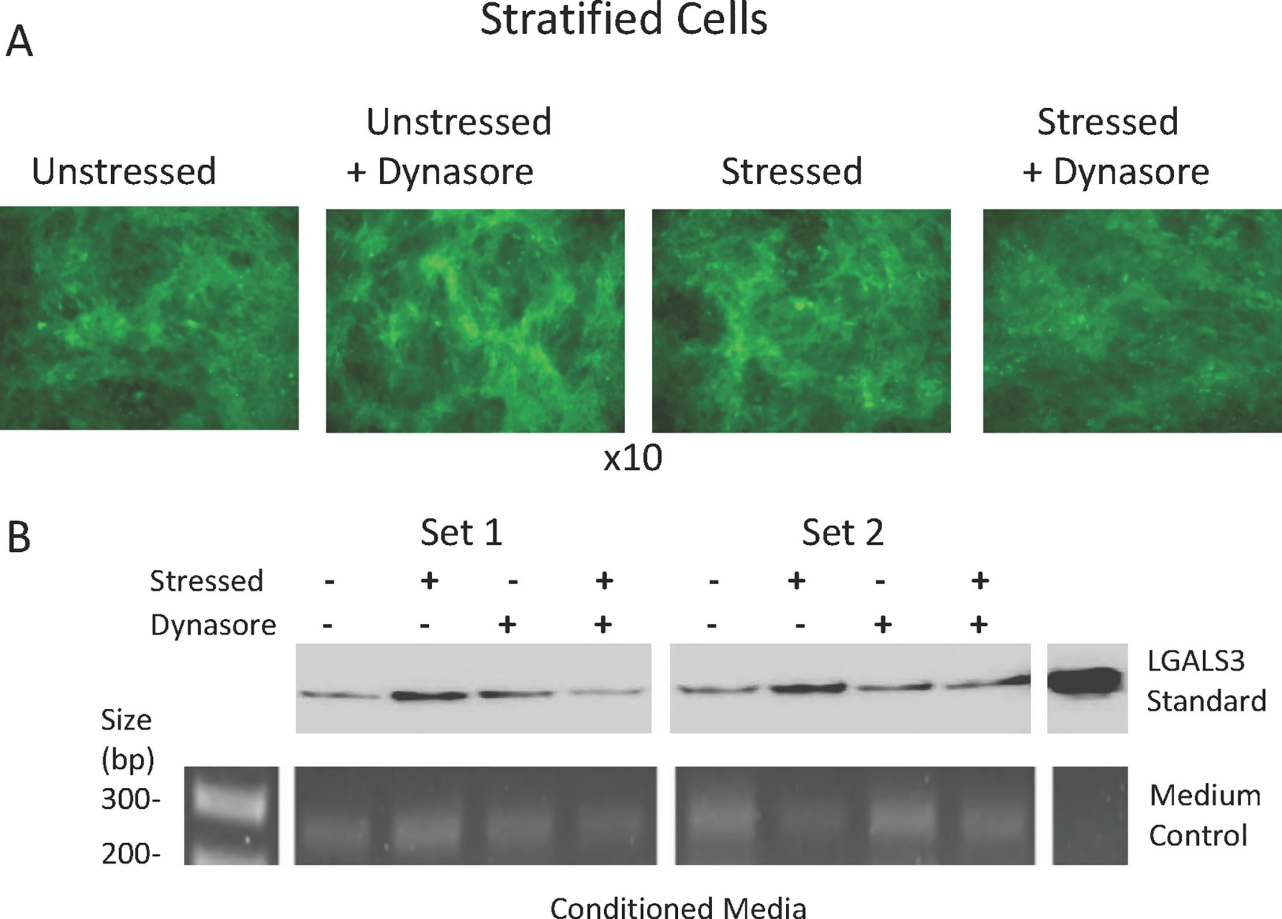
Evidence that Dynasore protects the cell surface glycocalyx against damage due to oxidative stress. HCLE stratified cultures plated in triplicate were either left unstressed or stressed with 3 mM tBHP. At the same time, a set of unstressed and stressed cultures were treated with Dynasore (40 uM). (An equal volume of the Dynasore diluent, DMSO, was added to untreated cultures.) After 2 hours, **(A)** the cells were stained with a lectin, fluorescein-conjugated jacalin and imaged under epifluorescent illumination. Representative images are shown from each triplicate set. **(B)** Proteins in the media were resolved by SDS-PAGE, western blotted, and probed with an antibody specific to LGALS3 (top). DNA in the medium was also subjected to genomic PCR for ACTB gene detection (bottom).

Next, we investigated protection of the glycocalyx, using stratified cell cultures with mucosal differentiation. Representative results are shown in Fig 6.

A fluorescently-labelled lectin binding assay was used to assess damage to the mucins at the surface of differentiated cells. Jacalin is a lectin that binds to O-linked glycans found on membrane-associated mucins of the ocular surface [42, 43]. Jacalin bound strongly to the ocular surface of unstressed cells, indicative of mucosal differentiation. However, no staining differences were observed between unstressed and stressed cells, and treatment with Dynasore also had no effect (Fig 6A). Thus, if oxidative stress damages the mucin-type glycoconjugates of the glycocalyx, the damage must be subtle and below the level of detectability by this method.

While we were unable to detect any changes using the lectin assay, damage to the glycocalyx was evident when LGALS3 was examined by Western blotting. Oxidative stress caused an increase in LGALS3 in the cell culture medium consistent with an increase in shedding from the apical cell surface; treatment with Dynasore inhibited this increase (Fig 6B, compare 2^nd^ lane to 4^th^ lane in set 1 and set 2). Levels of genomic DNA in the culture medium remained unaffected, consistent with an increase in LGALS3 shedding from the glycocalyx itself, rather than greater shedding of cells containing LGALS3 on their cell surface.

These results document cell and glycocalyx damage due to oxidative stress, show that treatment with Dynasore prevents damage to cells, and provide evidence that Dynasore also prevents damage to the cell surface glycocalyx.

## Discussion

Vital dyes are commonly used clinically to evaluate health of the ocular surface; however, staining mechanisms remain poorly understood. Recent evidence suggests that sublethal damage stimulates vital dye uptake by individual living cells. Since cell damage can also stimulate reparative plasma membrane remodeling, we hypothesized that dye uptake occurs via endocytic vesicles. In support of this idea, we show here that application of oxidative stress to relatively undifferentiated monolayer cultures of human corneal epithelial cells stimulates both dye uptake and endocytosis, and that dye uptake is blocked by co-treatment with three different endocytosis inhibitors. Stressing of stratified and differentiated corneal epithelial cell cultures, which are a better model of the ocular surface, also stimulated dye uptake; however, endocytosis was *not* stimulated, and two of the endocytosis inhibitors did not block dye uptake. The exception was Dynasore and its more potent analogue Dyngo-4a. Significantly, while Dynasore blocked oxidative stress-stimulated dye uptake at the ocular surface of *ex vivo* mouse eyes when treatment was performed at the same time as eyes were stressed, it had no effect when used *after* stress was applied and the ocular surface was already damaged. Thus, Dynasore could not be working by inhibiting endocytosis. Employing cytotoxicity and western blotting assays, we went on to demonstrate an alternative mechanism. We found that Dynasore is remarkably protective of cells and their surface glycocalyx, preventing damage due to stress, and thus precluding barrier disruption and dye entry.

Our results emphasize the importance of using stratified and differentiated cell cultures when modelling events at the ocular surface. Human corneal epithelial cells in monolayer culture are relatively undifferentiated, resembling basal cells of the corneal epithelium. However, when these cells are transferred to differentiation medium containing high calcium and left for 7 days, they develop stratified cell mounds that express markers of the mucosal glycocalyx, with the upper cell layer exhibiting a flattened morphology similar to the apical layer of the corneal epithelium [33]. In the current study, we observed differences in the appearance of oxidative stress-stimulated vital dye uptake in monolayer and stratified cells, with no cytoplasmic concentration in the latter. Our finding that oxidative stress does *not* stimulate endocytosis in stratified and differentiated cell cultures are consistent with the concept that stratified mucosal epithelia such as those of the ocular surface limit endocytosis as part of the barrier function to prevent passage of microbes and foreign antigens [47].

If endocytosis is not the mechanism for oxidative stress-stimulated vital dye uptake by cells at the ocular surface, then what is? A clue is provided by the our results using the trypan blue exclusion assay, which is based on the principle that live cells possess intact cell membranes that exclude certain dyes such as trypan blue, eosin, or propidium, whereas dead cells do not [41]. Recently it was shown that trypan blue staining does not necessarily indicate cell lysis, but may rather indicate pore formation in the cell membranes and more generally increased membrane permeability [48]. Trypan blue has also been shown recently to be a vital dye similar to fluorescein and rose bengal, with potential clinical applications [49]. Oxidative stress causes lipid peroxidation of the plasma membrane. Eventually, pore formation can occur [50], creating passages that are not large enough to kill cells, but that allow fluorescein to penetrate. Rose bengal should be excluded by the glycocalyx, but we report evidence here that glycocalyx barrier function is also altered by oxidative stress. This change also appears to be subtle, as it could not be detected by the lectin binding assay, however we did observe an increase in LGALS3 released into the cell culture medium. Dynamins are known to be involved in the conventional secretory pathway [51], however, LGALS3, like other members of the galectin family, lacks both a membrane-anchoring domain and a signal sequence. Instead of being transferred into the endoplasmic reticulum and Golgi compartments for classical secretion, LGALS3 is synthesized on clusters of free ribosomes in the cytoplasm of the cell as an non-glycosylated protein before secretion [52, 53]. Thus, it seems unlikely that Dynasore is acting to inhibit LGALS3 secretion. Taken together, the results suggest that no specific mechanism is needed for vital dye uptake; dye may simply enter the oxidative stress-damaged cell more easily because of small breeches in the plasma membrane and glycocalyx barriers. We suggest that the requirement for the cell to be alive for hyperfluorescence may be that active mechanisms are needed to concentrate the dye, and keep it from leaving the cell once it has entered.

A caveat to keep in mind is that we did not test multiple forms of stress in this study, therefore we cannot be entirely certain that findings using the oxidative stress model translate to other forms of stress. However, we note that results of our initial experiments to characterize the oxidative stress model were entirely consistent with those of the Bandamwar reference study that *did* test multiple forms of stress [20]. Whether SICS involves cell damage is still under debate. MPS characteristically contain a surfactant cleaner such as Tetronic 1107 [54], **a** biocide such as polyhexamethylene biguanide (which may also have surfactant properties [55]), and a buffering agent. Surfactants dissolve plasma membrane lipids while this may not cause significant toxicity we suggest that barrier function of the plasma membrane may be disrupted sufficiently to allow fluorescein to enter cells more freely. In addition, our group has shown that treatment of stratified and differentiated HCLE cell cultures with different MPS causes an increase in rose bengal staining similar to what we show here for oxidative stress, and we provided evidence for the associated shedding of MUC16 ectodomains [44].

How Dynasore and its analogue Dyngo-4a are so effective in protecting the ocular surface epithelial cells against oxidative stress remains an open and very intriguing question. These compounds were developed to specifically target dynamin family GTPases as an aid for the study of endocytosis [32]; however, other dynamin-dependent effects have been demonstrated. For example, targeting by Dynasore of dynamin family members DNM2 or DRP1 protects cardiomyocyte against entering apoptosis following oxidative stress, by alleviating mitochondrial fragmentation [56–58] and blocking inflammasome activation and inflammatory cytokine expression [59]. Dynasore also has dynamin-independent “off-target” effects on plasma membrane cholesterol, lipid rafts, and actin dynamics [60]. Both of these mechanisms might explain the protective effects of Dynasore on plasma membrane permeabilization and shedding of cell surface glycoproteins due to damaging oxidative stress, and will be important to investigate in future studies. To determine whether dynamin-dependent or -independent mechanisms are involved would require knockdown of individual dynamins, as was done in the cardiomyocyte studies [56–59].

After we submitted this manuscript for review, we became aware of a new publication by the same team that conducted one of the studies that served as a reference here [21]. Again, a model of monolayer cells in culture was used to investigate the mechanisms of fluorescein dye uptake in SICS [61]. It was found that MPS-stimulated fluorescein uptake was not associated with apoptosis, unlike the findings of our second reference study that examined damaging stress-stimulated fluorescein uptake [20], confirmed here for oxidative stress. Moreover, fluorescein uptake in cells treated with MPS was not associated with metabolic compromise, as we show here for oxidative stress. The surfactant Tectronic 1107 alone caused the same effects as the effective MPS. Nevertheless, Dynasore inhibited uptake of fluorescein in MPS-treated cells, similar to our findings in the current study using oxidative stress. The authors proposed that dynamin is directly involved in entry of fluorescein into MPS-treated monolayer cells in a mechanism that may involve encapsulation by polymeric micelles and endocytosis. It will be interesting to learn whether this conclusion holds in the stratified and differentiated cell cultures that more closely model the ocular surface.

Dynasore has been proposed as a candidate therapeutic to treat diseases involving abnormal mitochondrial dynamics [62, 63], and our results presented here suggest that it might also be valuable to prevent ocular surface disease. Moreover, while we observed that Dynasore and Dyngo-4a had no effect on vital dye uptake once the damage was done, they would still have value for treatment for ongoing disease. As discussed in the Introduction to this article, the ocular surface epithelia are constantly and rapidly turning over, with new cells rising up in the layers as the apical cells are desquamated. If Dynasore or Dyngo-4a are applied topically over a period of time, the new cells that rise to the surface would be protected. Thus, we predict that chronic ocular surface disease could gradually be resolved by such treatment.

## Conclusions

Here we made the unexpected and novel discovery that Dynasore and its more potent analog Dyngo-4a protect ocular surface epithelial cells and their glycocalyx against damaging stress. These unexpected and novel findings provide greater insight into the mechanisms of vital dye uptake and point the direction for future study. Our results also suggest that Dynasore and its analogues might have therapeutic value in the treatment of ocular surface disease, and this use should be explored.
