# Correction: Dynasore protects the ocular surface against damaging oxidative stress

**DOI:** 10.1371/journal.pone.0208864

**Published:** 2018-12-05

**Authors:** The PLOS ONE Staff

## Notice of republication

An incomplete, earlier version of this article was published in error. The publisher apologizes for the error. This article was republished on November 19, 2018 to correct for this error. Please download the article again to view the correct version. The originally published, uncorrected article and the republished, corrected article are provided here for reference.

## Supporting information

S1 File Originally published, uncorrected article. (PDF)

S2 File Republished, corrected article. (PDF)
